# COVID-19 Bivalent Booster Vaccination Coverage and Intent to Receive Booster Vaccination Among Adolescents and Adults — United States, November–December 2022

**DOI:** 10.15585/mmwr.mm7207a5

**Published:** 2023-02-17

**Authors:** Peng-jun Lu, Tianyi Zhou, Tammy A. Santibanez, Anurag Jain, Carla L. Black, Anup Srivastav, Mei-Chuan Hung, Jennifer L. Kriss, Susanne Schorpp, David Yankey, Natalie Sterrett, Hannah E. Fast, Hilda Razzaghi, Laurie D. Elam-Evans, James A. Singleton

**Affiliations:** ^1^Immunization Services Division, National Center for Immunization and Respiratory Diseases, CDC; ^2^Leidos Inc., Atlanta, Georgia; ^3^Goldbelt C6, Chesapeake, Virginia; ^4^Oak Ridge Institute for Science and Education, Oak Ridge, Tennessee.

COVID-19 vaccine booster doses are safe and maintain protection after receipt of a primary vaccination series and reduce the risk for serious COVID-19–related outcomes, including emergency department visits, hospitalization, and death (*1**,**2*). CDC recommended an updated (bivalent) booster for adolescents aged 12–17 years and adults aged ≥18 years on September 1, 2022 (*3*). The bivalent booster is formulated to protect against the Omicron BA.4 and BA.5 subvariants of SARS-CoV-2 as well as the original (ancestral) strain (*3*). Based on data collected during October 30–December 31, 2022, from the National Immunization Survey–Child COVID Module (NIS-CCM) (*4*), among all adolescents aged 12–17 years who completed a primary series, 18.5% had received a bivalent booster dose, 52.0% had not yet received a bivalent booster but had parents open to booster vaccination for their child, 15.1% had not received a bivalent booster and had parents who were unsure about getting a booster vaccination for their child, and 14.4% had parents who were reluctant to seek booster vaccination for their child. Based on data collected during October 30–December 31, 2022, from the National Immunization Survey–Adult COVID Module (NIS-ACM) (*4*), 27.1% of adults who had completed a COVID-19 primary series had received a bivalent booster, 39.4% had not yet received a bivalent booster but were open to receiving booster vaccination, 12.4% had not yet received a bivalent booster and were unsure about getting a booster vaccination, and 21.1% were reluctant to receive a booster. Adolescents and adults in rural areas had a much lower primary series completion rate and up-to-date vaccination coverage. Bivalent booster coverage was lower among non-Hispanic Black or African American (Black) and Hispanic or Latino (Hispanic) adolescents and adults compared with non-Hispanic White (White) adolescents and adults. Among adults who were open to receiving booster vaccination, 58.9% reported not having received a provider recommendation for booster vaccination, 16.9% had safety concerns, and 4.4% reported difficulty getting a booster vaccine. Among adolescents with parents who were open to getting a booster vaccination for their child, 32.4% had not received a provider recommendation for any COVID-19 vaccination, and 11.8% had parents who reported safety concerns. Although bivalent booster vaccination coverage among adults differed by factors such as income, health insurance status, and social vulnerability index (SVI), these factors were not associated with differences in reluctance to seek booster vaccination. Health care provider recommendations for COVID-19 vaccination; dissemination of information by trusted messengers about the continued risk for COVID-19–related illness and the benefits and safety of bivalent booster vaccination; and reducing barriers to vaccination could improve COVID-19 bivalent booster coverage among adolescents and adults.

NIS-CCM and NIS-ACM data were collected by telephone interview in English, Spanish, or other languages using a random-digit–dialed sample of cellular telephone numbers. Data collected during October 30–December 31, 2022,* were analyzed to assess demographic, behavioral, and social factors associated with COVID-19 primary series vaccination,^†^^,^^§^^,^^¶^ bivalent booster receipt,**^,^^††^^,^^§§^ up-to-date COVID-19 vaccination status,^¶¶^ and, among adults or their children who had not received a bivalent booster dose, intent to receive booster vaccination or to get their child a booster vaccination. Receipt of an updated bivalent booster was not explicitly asked of respondents; however, only bivalent boosters were authorized after September 1, 2022 (*3*). Thus, a booster vaccination received after September 1, 2022, was assumed to be a bivalent booster. The cumulative NIS-CCM and NIS-ACM response rates as of December 2022 were 18.2% and 23.2%, respectively. Bivalent booster dose receipt and intention to receive (or have child receive) a booster dose were assessed among the subset of respondents who had completed the primary COVID-19 vaccination series (*5*) (2,900 [NIS-CCM]; 83,462 [NIS-ACM]). Primary series completion and up-to-date COVID-19 vaccination status were assessed among all adolescents (4,383 [NIS-CCM]) and adults (99,056 [NIS-ACM]).

Primary series completion, up-to-date COVID-19 vaccination status, bivalent booster vaccination status, and intention to receive (or have one’s child receive) a booster were stratified by race and ethnicity,*** metropolitan statistical area (MSA),^†††^ SVI,^§§§^ other demographic characteristics, and behavioral and social drivers of vaccination (*6*). Persons considered open to booster vaccination included those who reported they definitely or probably would get booster vaccination for themselves or their child. Persons considered reluctant to receive booster vaccination included those who reported they probably or definitely would not get a booster for themselves or their child. Data were analyzed using SAS (version 9.4; SAS Institute) and SUDAAN (version 11.0.1; Research Triangle Institute). All percentages were weighted to represent the noninstitutionalized U.S. adolescent or adult population.^¶¶¶^ T-tests were used to determine differences between groups with p<0.05 considered statistically significant. This activity was reviewed by CDC and was conducted consistent with applicable federal law and CDC policy.****

From interviews conducted during November–December 2022, 58.3% of all adolescents aged 12–17 years had completed a COVID-19 vaccine primary series, and 10.7% were up to date with COVID-19 vaccination (Table 1). Among adolescents who had completed a COVID-19 primary series, 18.5% had received a bivalent booster since September 1, 2022; 52.0% had not received a bivalent booster but had parents who were open to booster vaccination for their child (31.0% definitely would and 21.0% probably would), 15.1% had parents who were unsure about getting a booster vaccination for their child, and 14.4% of adolescents had parents who were reluctant to get a booster vaccination for their child. Up-to-date COVID-19 vaccination status among all adolescents, and bivalent booster coverage among those who had completed the primary series, increased from November (9.2% and 15.7%, respectively) to December (12.3% and 21.3%, respectively). COVID-19 primary series completion was similar among Black and White adolescents and higher among Hispanic and non-Hispanic Asian (Asian) adolescents compared with White adolescents, while bivalent booster coverage was lower among Black (10.2%), Hispanic (14.6%), and non-Hispanic other or multiracial adolescents (14.0%) than among White (22.9%) adolescents. Adolescents who were uninsured and those living in a high SVI county had lower bivalent booster coverage compared with those who were insured and living in lower SVI counties. Reluctance to seek child’s booster vaccination was lower among Hispanic compared with White adolescents’ parents; however, reluctance to seek booster vaccination did not differ by insurance or SVI status. Adolescents in rural (non-MSA) areas had lower COVID-19 vaccine primary series completion rate and up-to-date coverage than those in MSA principal city areas. 

**TABLE 1:** COVID-19 primary vaccine series completion and up-to-date COVID-19 vaccination status* among all adolescents aged 12–17 years and bivalent booster vaccination coverage among those who had completed the primary COVID-19 vaccination series, by demographic and behavioral characteristics — National Immunization Survey–Child COVID Module, United States, October 30–December 31, 2022

Characteristic	Total no.	%^†^ (95% CI)		Adolescents who completed primary series of COVID-19 vaccine				
		Adolescents who completed primary COVID-19 vaccination series	Up to date with COVID-19 vaccination*	No.	%^†^ (95% CI)			
					Bivalent booster coverage among those with completed primary series	Parental intent to get booster dose for child		
						Definitely or probably will	Unsure	Definitely or probably will not
**Total**	**4,383**	**58.3 (55.7–60.8)**	**10.7 (9.5–12.2)**	**2,900**	**18.5 (16.3–20.9)**	**52.0 (48.8–55.2)**	**15.1 (12.9–17.6)**	**14.4 (12.2–16.9)**
**Month of interview**								
Nov^§^	**1,992**	58.2 (54.3–62.0)	9.2 (7.5–11.1)	1,328	15.7 (12.9–19.1)	54.6 (49.6–59.5)	15.4 (12.1–19.4)	14.2 (10.9–18.4)
Dec	**2,391**	58.3 (54.9–61.7)	12.3 (10.4–14.5)^¶^	1,572	21.3 (18.2–24.8)^¶^	49.3 (45.3–53.4)	14.9 (12.1–18.1)	14.5 (11.8–17.8)
**Age group, yrs**								
12–14^§^	**2,656**	51.8 (48.2–55.4)	10.3 (8.6–12.2)	1,654	19.3 (16.1–22.8)	54.1 (49.4–58.7)	14.3 (11.0–18.4)	12.4 (9.8–15.4)
15–17	**1,727**	64.8 (61.1–68.4)^¶^	11.2 (9.4–13.4)	1,246	17.9 (14.9–21.2)	50.3 (45.9–54.7)	15.8 (13.0–19.1)	16.1 (12.7–20.1)
**Sex**								
Female	**2,063**	60.3 (56.6–63.9)	10.9 (9.1–13.1)	1,372	19.2 (16.0–22.8)	50.5 (45.7–55.3)	15.2 (12.0–19.1)	15.1 (11.7–19.1)
Male^§^	**2,290**	56.3 (52.7–59.8)	10.5 (8.7–12.5)	1,508	17.6 (14.7–20.9)	53.4 (49.1–57.6)	15.2 (12.3–18.6)	13.8 (11.0–17.3)
**Race and ethnicity**								
Asian, non-Hispanic	**232**	88.3 (79.4–93.7)^¶^	15.5 (9.5–24.3)	195	18.2 (11.0–28.6)	57.1 (42.5–70.5)	10.7 (5.5–19.9)**	14.0 (6.6–27.3)**
Black or African American, non-Hispanic	**407**	54.2 (46.8–61.6)	5.6 (3.1–10.0)^¶^	261	10.2 (5.5–18.2)^¶,^**	52.3 (43.1–61.3)	20.4 (14.2–28.4)	17.1 (11.3–25.0)
Hispanic or Latino	**858**	64.3 (58.1–70.0)^¶^	8.7 (6.2–12.1)^¶^	568	14.6 (10.4–20.0)^¶^	57.3 (50.2–64.1)^¶^	18.1 (13.5–23.9)	10.0 (6.7–14.7)^¶^
White,^§^ non-Hispanic	**2,500**	54.5 (51.2–57.7)	12.4 (10.7–14.5)	1,630	22.9 (19.8–26.4)	47.9 (43.9–51.9)	13.5 (10.9–16.6)	15.7 (12.5–19.6)
Other (including AI/AN) and multiple races, non-Hispanic	**338**	57.4 (47.3–67.0)	10.4 (6.3–16.7)	220	14.0 (8.3–22.6)^¶^	58.9 (44.3–72.1)	14.8 (5.3–34.8)**	12.3 (6.2–22.8)**
**Urbanicity**								
MSA, principal city^§^	**1,319**	63.7 (58.8–68.3)	11.9 (9.5–14.8)	928	18.9 (15.1–23.3)	53.4 (47.6–59.1)	17.0 (13.4–21.3)	10.7 (7.9–14.5)
MSA, nonprincipal city	**2,219**	59.9 (56.3–63.4)	11.1 (9.4–13.1)	1,479	18.8 (16.0–22.1)	51.3 (47.0–55.6)	12.6 (9.8–16.1)	17.3 (13.9–21.3)^¶^
Non-MSA	**676**	37.4 (31.8–43.4)^¶^	6.9 (4.4–10.7)^¶^	347	16.9 (10.9–25.5)	50.8 (41.4–60.2)	23.0 (15.5–32.9)	9.2 (5.6–14.8)
**SVI of county of residence^††^**								
Low^§^	**1,589**	61.7 (57.5–65.7)	14.4 (12.0–17.1)	1,101	23.3 (19.6–27.5)	49.5 (44.8–54.2)	12.5 (9.6–16.1)	14.7 (11.4–18.7)
Moderate	**1,477**	59.0 (54.6–63.2)	11.5 (9.3–14.0)	969	19.4 (15.8–23.6)	54.7 (49.4–60.0)	13.1 (10.1–16.8)	12.8 (9.5–17.1)
High	**1,077**	53.8 (48.7–58.8)^¶^	7.0 (5.1–9.6)^¶^	653	13.2 (9.5–18.0)^¶^	51.4 (44.6–58.2)	20.8 (15.6–27.1)^¶^	14.6 (10.1–20.6)
**Household income**								
Below poverty level^§^	**438**	45.6 (37.8–53.6)	8.1 (4.9–13.2)	249	17.6 (10.7–27.7)	48.6 (37.9–59.5)	19.5 (13.1–28.0)	14.2 (6.9–27.0)**
Above poverty level, <$75,000	**953**	47.0 (41.9–52.1)	7.8 (5.5–10.9)	539	14.8 (10.3–20.7)	53.0 (46.0–59.9)	17.5 (13.1–23.1)	14.7 (10.4–20.5)
Above poverty level, ≥$75,000	**2,150**	67.2 (63.5–70.6)^¶^	14.7 (12.6–17.1)^¶^	1,551	22.7 (19.5–26.2)	53.2 (48.8–57.6)	10.7 (8.4–13.5)^¶^	13.4 (10.6–16.8)
Unknown	**842**	62.1 (56.3–67.5)^¶^	7.2 (5.2–9.9)	561	12.0 (8.6–16.5)	49.7 (42.2–57.2)	21.6 (15.0–30.0)	16.7 (11.8–23.2)
**Mother's education level**								
High school diploma or less^§^	**906**	49.0 (43.6–54.4)	7.2 (5.0–10.1)	499	13.9 (9.7–19.5)	53.9 (46.6–61.1)	16.9 (12.5–22.5)	15.3 (10.4–22.0)
Some college	**1,071**	46.7 (42.1–51.4)	6.9 (5.0–9.5)	597	13.4 (9.6–18.5)	52.5 (45.7–59.3)	19.5 (14.1–26.3)	14.6 (10.6–19.8)
College degree or more	**2,327**	73.1 (69.6–76.5)^¶^	16.2 (14.0–18.6)^¶^	1,765	23.4 (20.3–26.8)^¶^	50.8 (46.6–55.0)	12.2 (9.7–15.1)	13.6 (10.8–17.0)
**Health insurance**								
Medicaid	**1,174**	47.9 (43.2–52.6)	7.5 (5.5–10.1)^¶^	657	15.2 (11.1–20.5)^¶^	50.1 (43.5–56.7)	19.8 (14.9–26.0)	14.8 (10.5–20.5)
Other	**2,988**	65.6 (62.5–68.6)^¶^	13.5 (11.8–15.4)^¶^	2,120	20.8 (18.2–23.6)^¶^	52.5 (48.7–56.2)	12.4 (10.2–14.9)	14.4 (11.8–17.4)
Not insured^§^	**134**	46.2 (33.2–59.9)	0.4 (0.1–1.1)	68	1.0 (0.3–2.8)**	55.0 (34.6–73.9)**	29.8 (14.0–52.5)**	14.3 (5.3–33.1)**
**HHS region^§§^**								
Region 1^§^	**438**	80.1 (73.0–85.6)	23.7 (18.0–30.5)	340	32.1 (24.6–40.6)	44.1 (36.0–52.4)	13.9 (8.6–21.6)	10.0 (5.9–16.3)
Region 2	**414**	66.1 (57.4–73.9)^¶^	7.0 (4.3–11.3)^¶^	308	11.9 (7.3–18.7)^¶^	48.7 (39.7–57.8)	16.1 (10.6–23.9)	23.2 (16.3–32.1)^¶^
Region 3	**757**	65.2 (60.0–70.1)^¶^	13.0 (10.2–16.5)^¶^	543	20.3 (16.1–25.4)^¶^	56.2 (50.3–61.9)^¶^	13.0 (9.6–17.4)	10.5 (7.4–14.6)
Region 4	**520**	46.7 (40.8–52.8)^¶^	6.8 (4.5–10.3)^¶^	307	13.7 (8.9–20.5)^¶^	52.2 (44.0–60.2)	16.3 (11.0–23.5)	17.9 (12.2–25.4)
Region 5	**582**	48.0 (42.1–53.9)^¶^	12.4 (9.3–16.3)^¶^	385	26.0 (20.0–33.2)	51.1 (43.9–58.3)	13.4 (9.1–19.3)	9.5 (6.3–13.9)
Region 6	**504**	63.8 (58.1–69.2)^¶^	11.0 (7.6–15.8)^¶^	284	16.2 (10.9–23.4)^¶^	52.0 (43.9–60.0)	18.6 (12.9–26.0)	13.2 (8.6–19.7)
Region 7	**210**	48.2 (38.9–57.7)^¶^	11.7 (7.6–17.7)^¶^	138	23.8 (15.5–34.6)	55.6 (43.5–67.1)	9.4 (4.6–18.2)**	11.3 (5.0–23.5)**
Region 8	**431**	56.1 (48.7–63.3)^¶^	15.5 (10.9–21.6)	248	28.5 (20.6–38.0)	49.2 (40.1–58.4)	13.0 (7.6–21.3)	9.3 (5.4–15.5)
Region 9	**347**	66.6 (56.7–75.2)^¶^	9.9 (6.4–15.2)^¶^	228	14.6 (9.2–22.5)^¶^	54.8 (43.2–66.0)	15.1 (8.5–25.3)	15.5 (8.4–26.6)
Region 10	**180**	63.7 (52.5–73.6)^¶^	9.7 (5.0–18.1)^¶,^**	119	16.9 (8.7–30.2)^¶,^**	49.2 (35.6–62.9)	15.1 (7.5–28.1)**	18.8 (10.1–32.4)
**Interview language**								
English	**4,208**	58.1 (55.5–60.6)	11.0 (9.7–12.5)	2,785	18.9 (16.7–21.4)	51.5 (48.2–54.8)	14.6 (12.3–17.2)	14.9 (12.6–17.6)^¶^
Other language^§^	**175**	60.8 (45.0–74.6)**	6.8 (2.9–15.3)**	115	12.3 (5.4–25.7)**	58.4 (44.5–71.2)	22.8 (13.7–35.4)	6.4 (2.5–15.6)**
**Received influenza vaccination since July 1, 2022**								
Yes	**1,776**	77.0 (72.4–81.0)^¶^	26.8 (23.6–30.4)^¶^	1,421	35.4 (31.4–39.5)^¶^	44.0 (39.8–48.2)^¶^	11.5 (9.0–14.6)^¶^	9.2 (6.8–12.3)^¶^
No^§^	**2,539**	47.9 (44.7–51.2)	1.8 (1.3–2.5)	1,441	3.5 (2.5–5.0)	59.3 (54.5–63.9)	18.6 (15.2–22.7)	18.6 (15.1–22.7)
**Monovalent booster status among adolescents who completed primary series**								
Received ≥1 monovalent booster dose	**1,070**	100	26.0 (21.9–30.5)^¶^	985	27.9 (23.5–32.7)^¶^	59.0 (53.7–64.1)^¶^	8.0 (5.7–11.3)^¶^	5.1 (3.4–7.6)^¶^
Did not receive any monovalent booster^§^	**2,002**	100	14.4 (12.1–17.2)	1,915	13.5 (11.2–16.2)	48.3 (44.3–52.3)	18.9 (15.9–22.4)	19.3 (16.2–22.9)

**Abbreviations:** AI/AN = American Indian or Alaska Native; HHS = U.S. Department of Health and Human Services; MSA = metropolitan statistical area; SVI = social vulnerability index.

* Up-to-date COVID-19 vaccination status was defined as receipt of a primary COVID-19 vaccination series and ≥1 bivalent booster dose or, among those who had not received a bivalent booster, completion of the most recent COVID-19 vaccine dose (the most recent dose could be a primary dose or a monovalent booster dose) <2 months earlier.

^†^ Weighted.

^§^ Reference level.

^¶^ p<0.05 by T-test for comparisons of vaccination coverage within each variable with the indicated reference level.

** Proportion was based on sample size of >30 but did not meet National Center for Health Statistics’ reliability criteria (sample size [n<30] and/or CI half-width >15 and/or the relative CI width >130%).

^††^ The CDC and the Agency for Toxic Substances and Disease Registry SVI uses 15 U.S. Census Bureau variables to help officials identify communities that might need support before, during, or after disasters. https://www.atsdr.cdc.gov/placeandhealth/svi/index.html

^§§^ *Region 1*: Connecticut, Maine, Massachusetts, New Hampshire, Rhode Island, and Vermont; *Region 2*: New Jersey, New York, and Puerto Rico; *Region 3*: Delaware, District of Columbia, Maryland, Pennsylvania, Virginia, and West Virginia; *Region 4*: Alabama, Florida, Georgia, Kentucky, Mississippi, North Carolina, South Carolina, and Tennessee; *Region 5*: Illinois, Indiana, Michigan, Minnesota, Ohio, and Wisconsin; *Region 6*: Arkansas, Louisiana, New Mexico, Oklahoma, and Texas; *Region 7*: Iowa, Kansas, Missouri, and Nebraska; *Region 8*: Colorado, Montana, North Dakota, South Dakota, Utah, and Wyoming; *Region 9*: Arizona, California, Hawaii, and Nevada; *Region 10*: Alaska, Idaho, Oregon, and Washington.

Among adults aged ≥18 years interviewed during November–December 2022, 84.2% had completed a COVID-19 primary series, and 23.2% were up to date with COVID-19 vaccination (Table 2). Among adults who had completed a primary COVID-19 vaccination series, 27.1% had received a bivalent booster, 39.4% had not yet received the bivalent booster but reported being open to booster vaccination (23.1% definitely would and 16.3% probably would), 12.4% were unsure about getting a booster, and 21.1% were reluctant to get a booster. Up-to-date COVID-19 vaccination status among all adults, and bivalent booster coverage among those who had completed a primary series increased from November (21.0% and 24.4%, respectively) to December (25.4% and 29.7%, respectively). Primary COVID-19 vaccination series completion was similar among White, Black, and Hispanic adults and higher among Asian adults than among those of all other races and ethnicities. Bivalent booster dose coverage was lower among Black (21.2%), Hispanic (15.0%), and Asian (25.1%) adults compared with White adults (32.1%). Bivalent booster coverage was higher among adults who had received a provider recommendation for booster vaccination (34.9%) than among those without a provider recommendation (22.2%). Bivalent booster dose coverage among adults who lived below the poverty level, were uninsured, and who lived in a moderate or high SVI county was lower than coverage among their less economically disadvantaged and lower SVI counterparts, although reluctance to seek bivalent booster vaccination generally did not differ by poverty, insurance, or SVI status. Adults in rural (non-MSA) areas had lower COVID-19 vaccine primary series completion rate and up-to-date coverage than those in MSA principal city areas.

**TABLE 2:** COVID-19 primary vaccination series completion and up-to-date COVID-19 vaccination status* and bivalent booster vaccination coverage among adults aged ≥18 years who had completed the primary COVID-19 vaccination series, by demographic and behavioral characteristics — National Immunization Survey–Adult COVID Module, United States, October 30–December 31, 2022

Characteristic	Total no.	%^†^ (95% CI)		Adults who completed primary series				
		Completed primary COVID-19 vaccination series	Up to date with COVID-19 vaccination*	No.	%^†^ (95% CI)			
					Bivalent booster coverage among those with completed primary series	Intention to get a booster		
						Definitely or probably will	Unsure	Definitely or probably will not
**Total**	**99,056**	**84.2 (83.7–84.7)**	**23.2 (22.6–23.8)**	**83,462**	**27.1 (26.4–27.7)**	**39.4 (38.7–40.2)**	**12.4 (11.9–13.0)**	**21.1 (20.4–21.7)**
**Month of interview**								
Nov^§^	**40,495**	84.3 (83.6–85.1)	21.0 (20.2–21.9)	34,227	24.4 (23.4–25.4)	41.9 (40.7–43.1)	12.7 (11.9–13.5)	21.0 (20.0–22.0)
Dec	**58,561**	84.1 (83.4–84.8)	25.4 (24.6–26.2)^¶^	49,235	29.7 (28.8–30.6)^¶^	36.9 (36.0–37.9)^¶^	12.2 (11.5–12.9)	21.2 (20.3–22.0)
**Age group, yrs**								
18–49^§^	**44,936**	77.5 (76.6–78.3)	14.1 (13.5–14.8)	35,973	17.7 (17.0–18.5)	42.7 (41.6–43.8)	14.1 (13.4–14.9)	25.4 (24.4–26.4)
50–64	**26,919**	88.9 (88.0–89.7)^¶^	27.2 (26.0–28.5)^¶^	22,998	30.1 (28.8–31.6)^¶^	38.5 (37.0–40.1)^¶^	11.9 (10.9–13.1)^¶^	19.4 (18.2–20.7)^¶^
≥65	**25,572**	96.4 (95.7–96.9)^¶^	42.1 (40.5–43.6)^¶^	23,276	43.3 (41.7–44.9)^¶^	34.0 (32.5–35.6)^¶^	9.0 (8.1–10.0)^¶^	13.8 (12.7–14.9)^¶^
**Sex**								
Female	**51,060**	86.5 (85.8–87.2)^¶^	25.7 (24.8–26.5)^¶^	43,914	29.1 (28.2–30.1)^¶^	39.4 (38.3–40.5)	12.5 (11.8–13.3)	19.0 (18.1–19.8)^¶^
Male^§^	**47,031**	82.0 (81.2–82.8)	20.8 (20.0–21.6)	38,869	24.9 (24.0–25.9)	39.5 (38.4–40.6)	12.1 (11.4–12.9)	23.5 (22.5–24.5)
**Race and ethnicity**								
AI/AN, non-Hispanic	**1,070**	72.2 (65.8–77.9)^¶^	16.5 (11.8–22.6)^¶^	748	22.4 (16.2–30.3)^¶^	39.4 (32.2–47.1)	11.9 (8.2–17.0)	26.2 (20.3–33.1)
Asian, non-Hispanic	**4,871**	97.4 (96.4–98.1)^¶^	24.8 (21.9–27.9)	4,674	25.1 (22.1–28.2)^¶^	46.6 (43.1–50.1)^¶^	14.4 (12.0–17.2)^¶^	13.9 (11.7–16.4)^¶^
Black or African American, non-Hispanic	**10,558**	84.7 (83.1–86.2)	18.7 (17.0–20.5)^¶^	8,954	21.2 (19.2–23.3)^¶^	44.0 (41.5–46.5)^¶^	15.2 (13.5–16.9)^¶^	19.7 (17.7–21.8)
Hispanic or Latino	**12,574**	84.1 (82.7–85.5)	13.2 (12.0–14.5)^¶^	10,576	15.0 (13.6–16.5)^¶^	47.5 (45.3–49.7)^¶^	17.7 (16.0–19.5)^¶^	19.8 (18.1–21.6)^¶^
Native Hawaiian or other Pacific Islander, non-Hispanic	**520**	83.2 (74.4–89.4)	17.1 (10.6–26.4)^¶^	423	20.4 (12.4–31.5)^¶^	38.9 (27.7–51.5)	21.8 (13.0–34.2)^¶^	18.9 (7.7–39.5)**
White,^§^ non-Hispanic	**63,157**	83.8 (83.1–84.5)	27.3 (26.5–28.1)	53,341	32.1 (31.3–33.0)	36.1 (35.2–37.1)	10.0 (9.4–10.6)	21.7 (20.9–22.5)
Other and multiple races, non-Hispanic	**3,396**	77.3 (73.4–80.8)^¶^	18.5 (15.7–21.7)^¶^	2,580	23.7 (20.2–27.7)^¶^	38.2 (33.7–42.9)	11.7 (9.3–14.5)	26.4 (22.5–30.6)^¶^
**Urbanicity**								
MSA, principal city^§^	**36,639**	86.3 (85.4–87.1)	23.9 (22.9–24.9)	31,887	27.0 (25.9–28.2)	40.6 (39.2–42.0)	12.7 (11.7–13.7)	19.7 (18.6–20.9)
MSA, nonprincipal city	**46,994**	85.5 (84.8–86.2)	23.8 (23.0–24.6)	39,884	27.4 (26.5–28.3)	39.5 (38.4–40.5)	12.1 (11.4–12.9)	21.0 (20.2–21.9)
Non-MSA	**15,423**	74.2 (72.6–75.8)^¶^	19.4 (18.0–20.9)^¶^	11,691	25.7 (23.9–27.6)	36.1 (34.1–38.1)^¶^	13.1 (11.7–14.6)	25.2 (23.4–27.0)^¶^
**SVI of county of residence^††^**								
Low^§^	**29,005**	85.8 (84.8–86.7)	27.5 (26.5–28.6)	25,032	31.6 (30.5–32.8)	37.6 (36.4–38.9)	10.9 (10.0–11.9)	19.8 (18.8–20.9)
Moderate	**32,669**	85.5 (84.7–86.3)	24.6 (23.5–25.6)^¶^	27,643	28.3 (27.1–29.5)^¶^	40.1 (38.7–41.4)^¶^	11.3 (10.4–12.2)	20.4 (19.4–21.6)
High	**27,206**	82.4 (81.4–83.4)^¶^	20.1 (19.0–21.2)^¶^	22,756	23.9 (22.7–25.3)^¶^	41.3 (39.8–42.9)^¶^	13.1 (12.0–14.2)^¶^	21.7 (20.4–23.0)^¶^
**Household income**								
Below poverty level^§^	**8,615**	79.1 (77.2–80.9)	14.3 (12.8–16.0)	6,492	16.5 (14.7–18.5)	45.8 (43.1–48.5)	18.4 (16.4–20.5)	19.4 (17.3–21.6)
Above poverty level, <$75,000	**29,539**	83.3 (82.3–84.2)^¶^	21.7 (20.6–22.8)^¶^	24,507	25.6 (24.3–26.8)^¶^	41.7 (40.3–43.1)^¶^	11.5 (10.6–12.5)^¶^	21.2 (20.1–22.4)
Above poverty level, ≥$75,000	**40,320**	88.0 (87.2–88.7)^¶^	28.7 (27.7–29.7)^¶^	35,913	32.4 (31.3–33.5)^¶^	37.5 (36.4–38.7)^¶^	9.6 (8.8–10.5)^¶^	20.4 (19.5–21.5)
Unknown	**20,582**	81.7 (80.5–82.9)^¶^	20.6 (19.4–21.8)^¶^	16,550	24.5 (23.1–26.0)^¶^	36.7 (35.0–38.4)^¶^	16.1 (14.9–17.4)	22.7 (21.3–24.2)^¶^
**Education level**								
High school diploma or less^§^	**23,239**	77.2 (76.1–78.3)	15.6 (14.7–16.6)	17,134	19.5 (18.3–20.7)	40.6 (39.1–42.1)	16.4 (15.2–17.5)	23.6 (22.3–24.9)
Some college	**26,185**	84.3 (83.4–85.1)^¶^	22.2 (21.1–23.4)^¶^	21,065	26.0 (24.7–27.3)^¶^	39.7 (38.3–41.2)	11.7 (10.7–12.6)^¶^	22.6 (21.4–23.8)
College graduate	**46,772**	93.3 (92.8–93.8)^¶^	34.3 (33.3–35.3)^¶^	43,121	36.4 (35.3–37.4)^¶^	38.4 (37.3–39.5)^¶^	8.4 (7.7–9.0)^¶^	16.9 (16.1–17.8)^¶^
**Health insurance**								
Insured	**89,787**	86.1 (85.6–86.7)^¶^	25.1 (24.4–25.7)^¶^	77,084	28.6 (27.9–29.4)^¶^	39.0 (38.2–39.8)^¶^	11.7 (11.1–12.2)^¶^	20.7 (20.1–21.4)
Not insured^§^	**6,498**	67.2 (64.8–69.5)	7.7 (6.4–9.3)	4,334	10.5 (8.7–12.7)	47.2 (44.1–50.4)	18.9 (16.6–21.4)	23.4 (20.9–26.1)
**U.S.-born status**								
Non–U.S.-born	**12,947**	90.7 (89.5–91.8)^¶^	17.8 (16.3–19.3)^¶^	11,703	19.2 (17.6–20.9)^¶^	43.9 (41.8–46.1)^¶^	19.1 (17.4–20.9)^¶^	17.8 (16.2–19.5)^¶^
U.S.-born^§^	**81,491**	83.3 (82.7–83.9)	24.5 (23.8–25.1)	68,273	28.9 (28.2–29.7)	38.8 (37.9–39.6)	10.8 (10.2–11.4)	21.5 (20.8–22.3)
**HHS region^§§^**								
Region 1^§^	**9,433**	96.8 (96.1–97.3)	34.8 (33.0–36.7)	8,529	35.4 (33.6–37.3)	36.8 (34.8–38.7)	11.0 (9.7–12.4)	16.8 (15.4–18.5)
Region 2	**9,305**	95.9 (95.2–96.5)	25.0 (23.4–26.7)^¶^	8,410	25.6 (23.9–27.3)^¶^	38.1 (36.2–40.1)	13.2 (12.0–14.6)^¶^	23.1 (21.5–24.8)^¶^
Region 3	**19,392**	90.5 (89.5–91.3)^¶^	27.9 (26.6–29.2)^¶^	16,973	30.2 (28.8–31.6)^¶^	39.1 (37.5–40.6)	11.4 (10.4–12.5)	19.3 (18.1–20.6)^¶^
Region 4	**9,440**	79.5 (78.0–81.0)^¶^	17.3 (15.9–18.9)^¶^	7,438	21.1 (19.4–23.0)^¶^	40.5 (38.3–42.7)^¶^	13.2 (11.8–14.8)^¶^	25.1 (23.2–27.1)^¶^
Region 5	**14,124**	76.7 (75.0–78.2)^¶^	24.9 (23.4–26.4)^¶^	11,795	32.0 (30.2–34.0)^¶^	36.7 (34.8–38.6)	12.2 (10.8–13.8)	19.0 (17.5–20.7)
Region 6	**13,313**	78.3 (76.7–79.8)^¶^	16.7 (15.4–18.0)^¶^	10,716	20.6 (19.0–22.3)^¶^	43.3 (41.2–45.5)^¶^	14.1 (12.7–15.7)^¶^	21.9 (20.2–23.7)^¶^
Region 7	**4,221**	77.3 (75.0–79.4)^¶^	23.7 (21.5–25.9)^¶^	3,385	29.5 (27.0–32.2)^¶^	38.7 (36.0–41.4)	10.2 (8.6–12.2)	21.5 (19.4–23.8)^¶^
Region 8	**6,921**	82.7 (80.9–84.3)^¶^	23.8 (21.9–25.9)^¶^	5,340	28.3 (26.0–30.7)^¶^	38.6 (36.0–41.3)	11.1 (9.5–13.0)	21.9 (19.8–24.2)^¶^
Region 9	**9,895**	89.8 (88.6–90.9)^¶^	25.8 (23.9–27.7)^¶^	8,362	28.5 (26.5–30.6)^¶^	40.3 (37.9–42.7)^¶^	12.4 (10.9–14.1)	18.8 (17.0–20.8)
Region 10	**3,012**	85.8 (83.2–88.0)^¶^	27.0 (24.0–30.2)^¶^	2,514	31.4 (28.0–35.0)	39.1 (35.5–42.8)	10.4 (8.2–13.2)	19.1 (16.2–22.3)
**Interview language**								
English	**96,741**	84.3 (83.7–84.8)	23.8 (23.2–24.4)^¶^	81,638	27.8 (27.1–28.5)^¶^	39.0 (38.2–39.8)^¶^	11.7 (11.2–12.3)^¶^	21.5 (20.8–22.2)^¶^
Other language^§^	**2,315**	83.2 (80.1–85.9)	10.2 (8.0–12.9)	1,824	11.0 (8.6–14.0)	49.0 (44.6–53.5)	28.2 (24.3–32.5)	11.8 (9.2–15.0)
**Frontline and essential workers aged 18–64 yrs^¶¶^**								
Essential health care	**8,756**	90.3 (88.7–91.6)^¶^	23.5 (21.7–25.5)^¶^	7,929	25.4 (23.4–27.5)^¶^	37.3 (34.8–39.8)	12.0 (10.4–13.8)^¶^	25.4 (23.2–27.7)^¶^
School and child care	**2,904**	88.3 (85.0–91.0)^¶^	24.6 (21.3–28.3)^¶^	2,624	27.3 (23.7–31.3)^¶^	44.8 (40.4–49.2)^¶^	9.9 (8.0–12.2)^¶^	18.0 (14.9–21.6)^¶^
Other frontline worker	**4,461**	75.0 (72.4–77.5)	11.8 (10.0–13.7)	3,342	14.7 (12.5–17.1)	40.7 (37.4–44.0)	16.3 (13.6–19.4)	28.3 (25.4–31.5)
Other essential worker^§^	**8,726**	74.5 (72.6–76.4)	12.6 (11.2–14.1)	6,519	16.6 (14.8–18.6)	38.7 (36.2–41.3)	15.5 (13.7–17.6)	29.1 (26.8–31.6)
Persons who are not essential workers	**46,662**	81.3 (80.5–82.1)^¶^	18.9 (18.2–19.7)^¶^	38,363	22.8 (21.9–23.7)^¶^	42.4 (41.3–43.6)^¶^	13.1 (12.3–14.0)^¶^	21.7 (20.7–22.6)^¶^
Disability***								
Yes (any)	**10,191**	86.4 (85.0–87.8)^¶^	23.8 (21.9–25.7)	8,434	26.7 (24.6–28.9)	40.7 (38.4–43.1)	13.6 (11.9–15.4)	19.0 (17.1–21.0)^¶^
No^§^	**88,644**	84.0 (83.4–84.5)	23.1 (22.5–23.8)	74,876	27.1 (26.4–27.8)	39.3 (38.5–40.1)	12.3 (11.7–12.9)	21.3 (20.6–22.0)
**Received influenza vaccination since July 1, 2022**								
Yes	**46,760**	96.2 (95.7–96.6)^¶^	44.3 (43.3–45.4)^¶^	44,954	45.7 (44.6–46.8)^¶^	34.1 (33.1–35.2)^¶^	8.2 (7.5–8.8)^¶^	12.0 (11.3–12.8)^¶^
No^§^	**51,931**	75.2 (74.4–76.1)	7.4 (6.9–7.9)	38,217	9.2 (8.5–9.8)	44.5 (43.4–45.6)	16.6 (15.7–17.5)	29.8 (28.7–30.8)
**Reported medical conditions**								
Yes	**32,096**	90.1 (89.3–90.8)^¶^	32.7 (31.5–33.9)^¶^	28,645	35.8 (34.5–37.1)^¶^	40.4 (39.0–41.8)	9.7 (8.8–10.6)^¶^	14.1 (13.2–15.2)^¶^
No^§^	**65,830**	81.6 (80.9–82.3)	18.9 (18.3–19.6)	53,991	22.7 (21.9–23.5)	39.1 (38.1–40.0)	13.6 (13.0–14.3)	24.6 (23.8–25.4)
**Provider recommendation of the COVID-19 booster vaccine**								
Yes	**35,463**	97.0 (96.5–97.5)^¶^	34.4 (33.2–35.5)^¶^	34,627	34.9 (33.8–36.1)^¶^	42.3 (41.1–43.5)^¶^	9.3 (8.6–10.1)^¶^	13.5 (12.7–14.4)^¶^
No^§^	**63,593**	77.8 (77.1–78.6)	17.7 (17.0–18.3)	48,835	22.2 (21.4–23.0)	37.7 (36.7–38.6)	14.4 (13.7–15.2)	25.8 (24.9–26.7)
**Monovalent booster status among adults aged 18–49 years who completed primary series**								
Received ≥1 monovalent booster dose	**20,699**	100	26.2 (25.0–27.5)^¶^	20,647	26.3 (25.1–27.6)^¶^	49.6 (48.0–51.1)^¶^	11.6 (10.5–12.7)^¶^	12.6 (11.6–13.6)^¶^
Received no monovalent booster^§^	**15,384**	100	9.7 (8.8–10.7)	15,326	8.5 (7.7–9.5)	35.4 (33.8–37.0)	16.9 (15.7–18.1)	39.2 (37.6–40.8)
**Monovalent booster status among adults aged ≥50 years who completed primary series**								
Received ≥2 monovalent booster doses	**13,036**	100	51.4 (49.2–53.6)^¶^	13,003	51.5 (49.2–53.7)^¶^	41.1 (38.9–43.4)^¶^	4.6 (3.7–5.6)^¶^	2.9 (2.2–3.7)^¶^
Received 1 monovalent booster dose	**19,489**	100	40.0 (38.3–41.7)^¶^	19,420	40.2 (38.5–41.9)^¶^	37.0 (35.4–38.8)^¶^	9.7 (8.7–10.8)^¶^	13.0 (11.8–14.3)^¶^
Received no monovalent booster^§^	**13,970**	100	21.9 (20.3–23.7)	13,851	20.2 (18.6–21.9)	31.7 (29.9–33.6)	16.1 (14.6–17.8)	31.9 (30.1–33.8)

**Abbreviations:** AI/AN = American Indian or Alaska Native; HHS = U.S. Department of Health and Human Services; MSA = metropolitan statistical area; SVI = social vulnerability index.

* Up-to-date COVID-19 vaccination status was defined as receipt of a primary COVID-19 vaccination series and ≥1 bivalent booster dose or, among those who had not received a bivalent booster, completion of the most recent COVID-19 vaccine dose (the most recent dose could be a primary dose or a monovalent booster dose) <2 months earlier.

^†^ Weighted.

^§^ Reference level.

^¶^ p<0.05 by T-test for comparisons of vaccination coverage within each variable with the indicated reference level.

** Proportion was based on sample size of >30 but did not meet National Center for Health Statistics’ reliability criteria (sample size [n<30] and/or CI half-width >15 and/or the relative CI width >130%).

^††^ The CDC and the Agency for Toxic Substances and Disease Registry SVI uses 15 U.S. Census Bureau variables to help officials identify communities that might need support before, during, or after disasters. https://www.atsdr.cdc.gov/placeandhealth/svi/index.html

^§§^ *Region 1*: Connecticut, Maine, Massachusetts, New Hampshire, Rhode Island, and Vermont; *Region 2*: New Jersey, New York, and Puerto Rico; *Region 3*: Delaware, District of Columbia, Maryland, Pennsylvania, Virginia, and West Virginia; *Region 4*: Alabama, Florida, Georgia, Kentucky, Mississippi, North Carolina, South Carolina, and Tennessee; *Region 5*: Illinois, Indiana, Michigan, Minnesota, Ohio, and Wisconsin; *Region 6*: Arkansas, Louisiana, New Mexico, Oklahoma, and Texas; *Region 7*: Iowa, Kansas, Missouri, and Nebraska; *Region 8*: Colorado, Montana, North Dakota, South Dakota, Utah, and Wyoming; *Region 9*: Arizona, California, Hawaii, and Nevada; *Region 10*: Alaska, Idaho, Oregon, and Washington.

^¶¶^ Essential worker groups were categorized as essential healthcare personnel (including health care, social service, and death care workers), school and child care (including preschool or child care, K–12 school, and other schools and instructional settings), other frontline (including first response [e.g., police or fire protection], correctional facility, food and beverage store, agriculture, forestry, fishing, or hunting, food manufacturing facility, nonfood manufacturing facility, public transit, and United States Postal Service), other essential (including other essential that are not listed above), and not a frontline or essential worker (including those who were not employed).

*** Disability was defined as an affirmative response to the following survey question: “Do you have serious difficulty seeing, hearing, walking, remembering, making decisions, or communicating?”

Among all adults who had completed a COVID-19 primary series, 5.2% reported difficulty getting a booster vaccine, with a higher percentage of those open to booster vaccination (4.4%) and unsure about booster vaccination (6.6%) reporting difficulty than did those who were already vaccinated (3.6%) (Table 3). The most common barrier reported was difficulty getting an appointment (5.5% of adults). Overall, among all adults who had completed a COVID-19 vaccine primary series, Black, Hispanic, and non-Hispanic American Indian or Alaska Native adults were more likely to report difficulty getting a booster vaccine, and Black and Hispanic adults were less likely to report confidence about COVID-19 vaccination safety and receipt of provider recommendation for booster vaccination compared with White adults (Supplementary Table 1, https://stacks.cdc.gov/view/cdc/124394). Among adults who were open to or unsure about booster vaccination, 41.1% and 28.7%, respectively, received a provider recommendation for booster vaccination (58.9% and 71.3%, respectively, did not receive a provider recommendation), and 83.1% and 54.5% of adults, respectively, were confident about COVID-19 vaccination safety (16.9% and 45.5%, respectively, had safety concerns) (Table 3). Among adolescents with parents who were open to or unsure about booster vaccination for their children, 67.6% and 54.8% of these parents, respectively, received a provider recommendation for any COVID-19 vaccine for their child (32.4% and 45.2% did not receive a provider recommendation), and 88.2% and 54.4% of these adolescents, respectively, had parents who were confident about COVID-19 vaccination safety for their child (11.8% and 45.6%, respectively, had parents with safety concerns). Adults and parents of adolescents overwhelmingly reported that a COVID-19 vaccine is important (54%–98% across bivalent booster vaccination and booster vaccination intent categories). Over one half of adults and parents of adolescents reported that vaccination is important, even among those who were reluctance to seek a booster vaccine.

**TABLE 3:** Barriers to receiving COVID-19 booster vaccination among adults and attitudinal and social factors regarding COVID-19 vaccination among adults and adolescents, by bivalent booster vaccination and booster vaccination intent* among those who completed a COVID-19 vaccine primary series — National Immunization Survey–Adult COVID Module and National Immunization Survey–Child COVID Module, United States, October 30**–**December 31, 2022

Characteristic	%^†^ (95% CI)				
	Overall	Received COVID-19 bivalent booster vaccination	Definitely or probably will get booster	Unsure will get booster	Definitely or probably will not get booster
**Adults who completed primary COVID-19 vaccination series**					
**Total no.**	**83,462**	**27,340**	**31,240**	**8,944**	**15,938**
**Reported barriers in getting a booster vaccination among adults aged ≥18 years**					
Difficulty getting a booster vaccine (very or somewhat difficult)^§^	**5.2 (4.9–5.6)**	3.6 (3.1–4.2)	4.4 (3.9–4.9)^¶^	6.6 (5.6–7.8)^¶,^**	8.2 (7.2–9.2)^¶,^**
Difficulty getting an appointment	**5.5 (5.1–5.8)**	6.2 (5.5–6.9)	5.7 (5.2–6.3)	6.1 (5.0–7.3)	3.8 (3.1–4.6)^¶,^**
Difficulty knowing where to get vaccinated	**3.8 (3.5–4.1)**	2.5 (2.1–3.0)	4.0 (3.5–4.4)^¶^	5.7 (4.7–6.9)^¶,^**	4.1 (3.4–4.9)^¶^
Difficulty getting to vaccination sites	**3.0 (2.7–3.3)**	1.8 (1.5–2.2)	3.1 (2.7–3.6)^¶^	4.4 (3.5–5.5)^¶,^**	3.4 (2.7–4.4)^¶^
Vaccination sites not open at convenient times	**3.8 (3.5–4.2)**	2.6 (2.2–3.1)	3.9 (3.5–4.5)^¶^	5.2 (4.3–6.3)^¶,^**	4.4 (3.6–5.2)^¶^
Did not know whether eligible for a booster vaccine	**3.1 (2.8–3.4)**	2.5 (2.1–3.0)	3.5 (3.1–4.0)^¶^	3.4 (2.7–4.2)	2.8 (2.3–3.5)
Had a reaction to a previous dose of the COVID-19 vaccine	**3.1 (2.9–3.4)**	1.5 (1.2–1.8)	2.1 (1.8–2.4)^¶^	5.6 (4.5–6.9)^¶,^**	5.9 (5.0–6.8)^¶,^**
Difficulty with cost of getting a booster vaccine	**2.8 (2.6–3.1)**	0.8 (0.6–1.1)	3.1 (2.7–3.6)^¶^	4.3 (3.6–5.2)^¶,^**	4.0 (3.3–4.8)^¶,^**
**Attitudinal and social factors regarding COVID-19 vaccination among adults aged ≥18 years**					
Concerned about getting COVID-19 (very or moderately)^††^	**42.1 (41.3–42.9)**	56.4 (55.0–57.8)	47.1 (45.9–48.4)^¶^	34.1 (31.9–36.4)^¶,^**	19.0 (17.6–20.4)^¶,^**
Thinks a COVID-19 vaccine is important (very or somewhat)^††^	**86.7 (86.1–87.2)**	97.6 (97.1–98.0)	96.4 (95.9–96.9)^¶^	85.4 (83.7–86.9)^¶,^**	54.4 (52.6–56.1)^¶,^**
Thinks COVID-19 vaccine is safe (completely or very)^††^	**71.0 (70.2–71.7)**	87.4 (86.4–88.4)	83.1 (82.1–84.0)^¶^	54.5 (52.0–56.9)^¶,^**	33.5 (31.8–35.2)^¶,^**
Friends and family vaccinated (almost all or many)^††^	**83.2 (82.6–83.8)**	89.6 (88.6–90.5)	87.2 (86.3–88.0)^¶^	80.4 (78.5–82.2)^¶,^**	69.0 (67.3–70.7)^¶,^**
Provider recommendation of the COVID-19 booster vaccine	**38.4 (37.6–39.1)**	49.5 (48.0–50.9)	41.1 (39.9–42.4)^¶^	28.7 (26.6–30.8)^¶,^**	24.6 (23.1–26.1)^¶,^**
**Attitudinal and social factors regarding COVID-19 vaccination among parents of adolescents aged 12–17 years**					
**Total no.**	**2,900**	**591**	**1,536**	**392**	**381**
Concerned about getting COVID-19 vaccine for child (very or moderately)^††^	**39.5 (36.4–42.7)**	49.7 (43.1–56.3)	43.2 (38.8–47.8)	32.1 (25.3–39.9)^¶,^**	20.7 (14.6–28.5)^¶,^**
Thinks a COVID-19 vaccine is important for child (very or somewhat)^††^	**90.0 (87.9–91.8)**	97.6 (94.2–99.0)	97.2 (95.6–98.2)	83.9 (77.0–88.9)^¶,^**	60.5 (51.4–68.9)^¶,^**
Thinks COVID-19 vaccine is safe for child (completely or very)^††^	**76.7 (73.9–79.2)**	87.1 (82.1–90.9)	88.2 (85.3–90.6)	54.4 (46.4–62.2)^¶,^**	41.5 (32.5–51.0)^¶,^**
Friends and family had similar-aged children vaccinated (almost all or many)^††^	**73.3 (70.3–76.1)**	82.1 (76.1–86.8)	79.0 (75.2–82.3)	61.6 (53.2–69.4)^¶,^**	53.5 (44.2–62.6)^¶,^**
Received provider recommendation for the COVID-19 vaccine^††^	**65.6 (62.4–68.7)**	76.3 (70.2–81.4)	67.6 (63.3–71.7)^¶^	54.8 (46.0–63.3)^¶,^**	55.9 (46.6–64.9)^¶,^**

* For adolescents, booster vaccination intent represents reported parental intent to get a booster vaccine for their child.

^†^ Weighted percentage.

^§^ Respondents who had received a booster dose were asked, “How difficult was it for you to get a COVID-19 booster vaccine?” Respondents who had not received a booster dose were asked, “How difficult would it be for you to get a COVID-19 vaccine booster?”

^¶^ p<0.05 by T-test for comparisons with those who received bivalent booster vaccination as the reference level.

** p<0.05 by T-test for comparisons with those who have not received bivalent booster but will definitely or probably get bivalent booster as the reference level.

^††^ Questions were asked about COVID-19 vaccination generally and not specifically about COVID-19 booster dose vaccination.

## Discussion

From interviews conducted during November–December 2022, approximately 20% of adolescents aged 12–17 years and approximately 30% of adults who had completed a primary COVID-19 vaccination series had received a bivalent booster dose since it was recommended on September 1, 2022. However, a large percentage of adults and parents of adolescents reported intent to receive booster vaccination for themselves or their children, indicating that booster vaccination coverage could substantially increase with appropriate interventions tailored to these reachable populations.

Reduction in disparities in completion of primary COVID-19 vaccination by race and ethnicity likely contributed to a reduction in the disparities in COVID-19 age-adjusted mortality rates that were observed early in the pandemic (*7*). However, bivalent booster coverage was lower among Black and Hispanic adolescents and adults compared with White adolescents and adults. Tailored and community-led interventions that helped reduce racial and ethnic inequities in primary COVID-19 vaccination could help address reported racial and ethnic differences in barriers to and attitudes toward booster vaccination. These strategies include creating and training a network of local community-trusted messengers to address misinformation and promote accurate, culturally appropriate vaccine messaging; providing vaccination in additional settings such as churches, barbershops, mass vaccination sites, or community sites; and working with culturally competent health care providers to provide a recommendation for bivalent booster vaccination.^††††^^,^^§§§§^

Although bivalent booster vaccination coverage among adults differed by factors such as income, health insurance status, and SVI, these factors were not associated with differences in reluctance to seek booster vaccination. This finding suggests the presence of unmeasured structural or access barriers to vaccination, even though only a small percentage of adults who had not received a booster since September 1, 2022, reported difficulties associated with cost of getting a booster vaccine or getting to a vaccination site. Patterns among adolescents were similar, with those who were uninsured and living in high SVI areas having lower booster vaccination coverage, but similar parental reluctance to vaccinate their children compared with those with higher incomes and living in less vulnerable areas. Specific barriers to booster vaccination, such as financial barriers, were not assessed in parents of adolescents.

Findings from this study suggest that provider recommendation for a COVID-19 booster dose has a positive impact on receipt of bivalent booster vaccination. However, among adults who were open to vaccination or adolescents with parents open to vaccination, more than one half of adults and one in three parents of adolescents did not receive a provider recommendation. Those who were unsure about booster vaccination for themselves or their children, and thus also potentially reachable to be vaccinated, were even less likely to have received a provider recommendation. Safety concerns about vaccination were also prevalent among those open to or unsure about booster vaccination. Provider recommendations to all patients that include culturally appropriate communication about the benefits and safety of booster vaccination and dissemination of information about the safety of vaccine by other trusted messengers could improve COVID-19 vaccination coverage (*8*).

The findings in this report are subject to at least four limitations. First, response rates of the NIS-CCM and NIS-ACM were low (18% and 23%, respectively). Although survey weights were calibrated to COVID-19 vaccine administration data to mitigate possible bias from incomplete sampling frame, nonresponse, and misclassification of vaccination status, bias in estimates might remain after weighting. Second, COVID-19 vaccination was self-reported and might be subject to recall or social desirability bias. Third, respondents were not specifically asked about bivalent boosters, and all boosters received after September 1, 2022, were assumed to be bivalent boosters, which might have overestimated bivalent booster coverage if some persons had received a monovalent booster after September 1, 2022. Finally, the survey sampled noninstitutionalized U.S. adults via mobile telephone; therefore, adults who were incarcerated or nursing home residents might not be represented in the sample.

A large proportion of persons who have completed a primary COVID-19 vaccination series have not received the bivalent booster but are open to vaccination or have parents who are open to getting a booster vaccination for their child. Ongoing monitoring of intent to receive a booster vaccination (or to have one’s child vaccinated with the booster vaccine), barriers to vaccination, and differences in bivalent booster vaccination coverage by demographic factors will be helpful for improving and expanding tailored strategies to improve vaccination coverage. To improve coverage, communities should partner with medical providers, schools, and community organizations to administer bivalent booster vaccination onsite or provide a referral for vaccination, reduce barriers to receipt of vaccination, employ trusted messengers to discuss vaccine safety and effectiveness with adults or parents and guardians of adolescents, and emphasize the importance of staying up to date with their COVID-19 vaccination (*9**,**10*).

**Summary**

**What is already known about this topic?**

COVID-19 bivalent booster vaccination has been recommended for persons aged ≥12 years since September 1, 2022.

**What is added by this report?**

Based on interviews conducted during November–December 2022, only 27.1% of adults and 18.5% of adolescents who had completed a COVID-19 primary series received a bivalent booster, and coverage was lower among Black and Hispanic persons. An additional 39.4% of adults were open to booster vaccination, and an additional 52.0% of adolescents had parents who were open to booster vaccination for their children. Those in rural areas had much lower primary series completion rate and up-to-date vaccination coverage.

**What are the implications for public health practice?**

Health care provider recommendations for booster vaccination, dissemination of information about the safety of vaccine by trusted messengers, and reducing barriers to vaccination could improve COVID-19 booster vaccination coverage.

## Footnotes

* Approximates coverage as of November 30, 2022.

† COVID-19 vaccination status was based on responses to the questions, “Have you received at least one dose of a COVID-19 vaccine?,” “Which brand of COVID-19 vaccine did you receive for your first dose?,” “How many doses of a COVID-19 vaccine have you received?,” and “During what month and year did you receive your most recent COVID-19 vaccine?”

§ For adolescents aged 12–17 years, primary series completion was defined as completion of a 2-dose primary COVID-19 vaccine series.

¶ For adults, primary series completion was defined as receipt of a 2-dose primary mRNA or Novavax COVID-19 vaccine series for adults who are not immunocompromised or receipt of a 3-dose mRNA or Novavax COVID-19 vaccine series for adults who reported being immunocompromised. For respondents whose initial vaccine was Janssen (Johnson & Johnson) vaccine, primary series completion was defined as receipt of a single dose primary vaccine for adults who are not immunocompromised or receipt of 2-dose series for adults who reported being immunocompromised.

** For adolescents aged 12–17 years, bivalent booster dose was defined as, since September 1, 2022, the receipt of at least a third dose of COVID-19 vaccine after completion of a 2-dose primary series.

†† For adults, bivalent booster dose was defined as receipt of at least a third dose of COVID-19 vaccine since September 1, 2022, after completion of 2-dose primary mRNA or Novavax vaccine series for adults who are not immunocompromised or at least a fourth dose of the vaccine after completion of a 3-dose mRNA or Novavax vaccine series for adults who reported being immunocompromised.

§§ For adults whose initial vaccine was a Janssen vaccine, bivalent booster dose was defined as the receipt of at least a second dose of COVID-19 vaccine since September 1, 2022, for adults who are not immunocompromised or at least a third dose for adults who reported being immunocompromised.

¶¶ Up-to-date COVID-19 vaccination status was defined as receipt of a primary COVID-19 vaccination series and ≥1 bivalent booster dose or, among those who had not received a bivalent booster, completion of the most recent COVID-19 vaccine dose (the most recent dose could be a primary dose or a monovalent booster dose) <2 months earlier.

*** Those who reported Hispanic ethnicity were classified as Hispanic and could be of any race. For adults, “non-Hispanic other/multiple races” included non-Hispanic adults who reported “other” race or more than one race. For adolescents, “non-Hispanic other/multiple races” included non-Hispanic American Indian or Alaska Native, non-Hispanic Native Hawaiian or other Pacific Islander, and non-Hispanic other or multiple races.

††† Urbanicity status was derived based on the centroid of the zip code of residence, categorized as MSA principal city, MSA nonprincipal city, or non-MSA.

§§§ Categorization into an SVI level was based on respondent-reported zip code of residence. https://www.atsdr.cdc.gov/placeandhealth/svi/index.html

¶¶¶ Survey weights were also calibrated by age and sex to state-level vaccine administration data reported to CDC by jurisdictions as of the middle of the monthly data collection period. https://covid.cdc.gov/covid-data-tracker/ (Accessed November 29, 2022).

**** 45 C.F.R. part 46.102(l)(2), 21 C.F.R. part 56; 42 U.S.C. Sect. 241(d); 5 U.S.C. Sect. 552a; 44 U.S.C. Sect. 3501.

†††† COVID-19 vaccine equity for racial and ethnic minority groups. https://www.cdc.gov/coronavirus/2019-ncov/community/health-equity/vaccine-equity.html

§§§§ Partnering for vaccine equity. https://www.cdc.gov/vaccines/health-equity/index.html
